# A Case Delayed Hemorrhage from the Stump of the Superior Rectal Artery after Abdominoperineal Resection of the Rectum

**DOI:** 10.1155/2010/961703

**Published:** 2010-03-24

**Authors:** Yuichi Sanada, Kenichi Nonaka, Takao Takahashi, Yoshihiro Tanaka, Shinji Osada, Kazuhiro Yoshida

**Affiliations:** Department of Surgical Oncology, Gifu Graduate School of Medicine, 1-1 Yanagido, 501-1194 Gifu, Japan

## Abstract

A 66-year-old man underwent abdominoperineal resection for advanced rectal cancer. On day 3 post surgery, a decompression tube was placed for postoperative ileus. Symptoms associated with ileus immediately disappeared. On day 7 post surgery, the patient vomited large amounts of fresh blood and became hemodynamically unstable. An emergency angiography revealed active bleeding from the stump of the superior rectal artery communicating with the third portion of the duodenum. Complete obliteration of the stump by proximal coil embolization was performed to achieve successful hemostasis. The postclinical course was uneventful and the patient was discharged on day 40 post surgery.

## 1. Introduction

The ligation of the inferior mesenteric artery (IMA) or superior rectal artery (SRA) is required for the complete dissection of regional lymph nodes in rectal cancer. Here we report a case of delayed hemorrhage arising from the stump of the SRA after abdominoperineal resection for rectal cancer. Postoperative bleeding in colorectal surgery has been commonly reported in cases of hemorrhage arising from pseudoaneurysm of the pelvirectal vessels [1]. Our case is considered to be a quite rare complication.

## 2. Case Report

**Figure 1:**
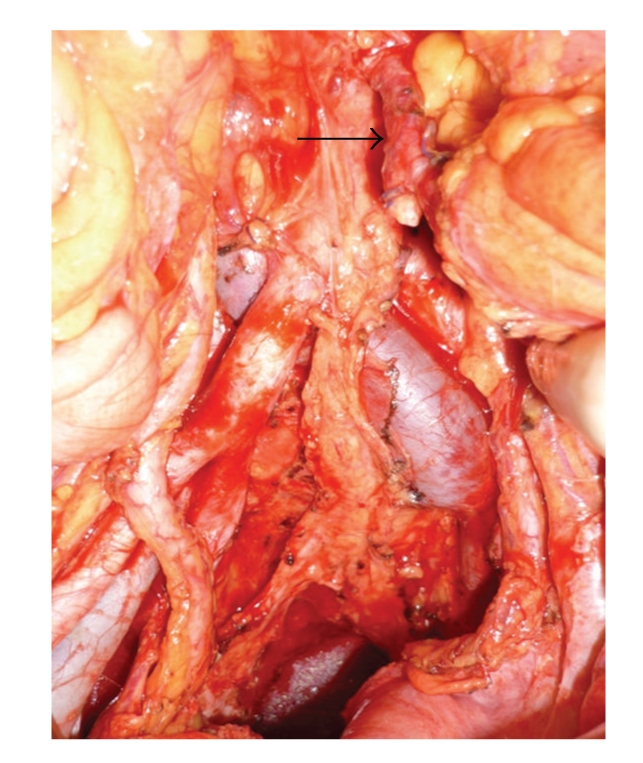
An intraoperative image after dissection of regional lymph nodes. The stump of the SRA is visible (arrow).

**Figure 2:**
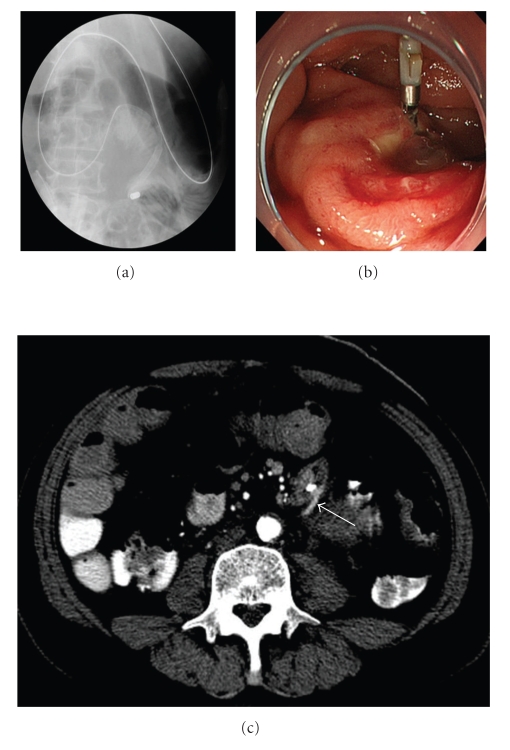
(a) A decompression tube was placed for postoperative ileus on day 3 post surgery. (b) Upper endoscopy shows an ulcerative lesion with pulsatile bleeding in the third portion of the duodenum. A marking clip was placed near the ulcer. (c) A computed tomography reveals that the marking clip and the stump of the SRA were very close (arrow).

**Figure 3:**
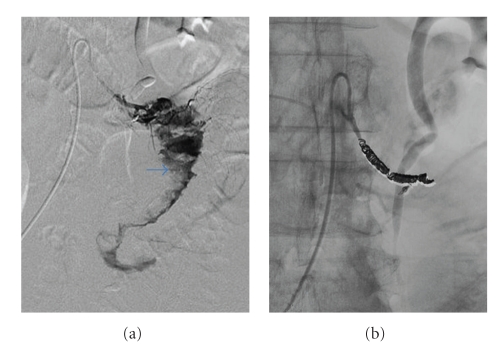
(a) An emergency angiography shows active bleeding from the stump of the SRA directly communicating with the third portion of the duodenum. (b) A complete embolization with microcoils was performed.

**Figure 4:**
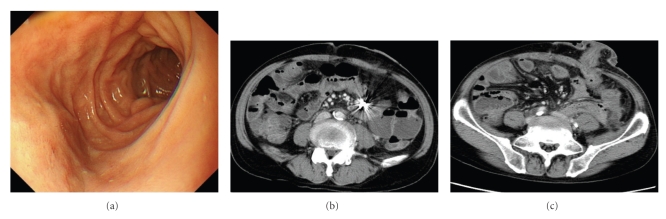
(a) Upper endoscopy on day 14 post coil embolization reveals neither ulceration nor stenosis of the third portion of the duodenum. (b and c) Computed tomography on day 14 post coil embolization. Intraabdominal abscess formation is not observed around the microcoils (b). The end colostomy was edematous and significantly affected by the obliteration of the left colic artery (c).

A 66-year-old man underwent abdominoperineal resection of the rectum for advanced rectal cancer after preoperative chemoradiotherapy. During the operation, the root of the inferior mesenteric artery (IMA) was not ligated. The IMA was tagged and preserved, separating the nervous and lymphatic tissues from the root to a site just peripheral of the confluence of the left colic artery (LCA), then the superior.rectal artery (SRA) was ligated (Figure 1). On day 3 post surgery, the patient experienced epigastric pain and vomited a large amount of bile-stained fluid. Plain abdominal roentgenograms showed marked gaseous distension of the upper small bowel. Under upper endoscopy, a decompression tube was placed at the upper jejunum 30 cm distal to the ligament of Treitz (Figure 2(a)). Because the third portion of the duodenum was bent caudally, the decompression tube could not be passed more distally. By day 5 post surgery, symptoms associated with ileus had disappeared. On the seventh postoperative day, the patient was awakened by epigastric pain, immediately followed by copious coffee-grounds diarrhea from the end colostomy. Although anemia and edema of the eyelids were present, the patient's general appearance was good. Upper endoscopy demonstrated a large amount of fresh blood in the stomach and a longuitudinal ulcerative lesion having a visible pulsative vessel in the base of the third portion of the duodenum (Figure 2(b)). Because of the severe pulsation and intermittent but spurting bleeding, hemostasis under endoscopy could not be performed. To identify the origin of bleeding, an emergency computed tomography (CT) was performed. Surprisingly, on arterial-phase CT revealed that the marking clip placed at the third portion of the duodenum was very close to the stump of the SRA (Figure 2(c), arrow). This finding implied that the source of bleeding was the stump of the SRA. Thirty minutes after the emergency CT, the patient vomited a large amount of fresh blood and became hemodynamically unstable. Immediately after fluids resuscitation and transfusion with 4 packed RBC and FFP, the patient was transferred to the angiography room. Emergency angiography revealed active projectile bleeding from the stump of the SRA (Figure 3(a)). The stump of the SRA was in direct communication with the third portion of the duodenum (Figure 3(a), arrow). Extraluminal hemorrhage was not identified. The length of the proximal portion of the IMA was considered to be sufficient for embolization. The stump of the SRA was obliterated using microcoils (TORNADO) between the root of the IMA and the tip of the ligation distal to the LCA (Figure 3(b)). Coil embolization was successful for achieving hemostasis. During the next 24 hours, the patient underwent fluid resuscitation and blood transfusion including 4 packs of RBC and FFP. Rebleeding did not develop after coil embolization. By the 10th day after coil embolization, symptomatic improvement and hemodynamically stable status without dopamine were achieved. On the 14th day after coil embolization, upper endoscopy revealed only the flexion of the third portion of the duodenum (Figure 4(a)). CT showed no sign of intraabdominal hemorrhage (Figure 4(b)). Edema and mild ischemic change of the end colostomy were identified as due to obstruction of the LCA arising from coil embolization. A diet was given from the 15th day after coil embolization. The clinical course was uneventful and the patient was discharged on the 40th postoperative day.

**Figure 5:**
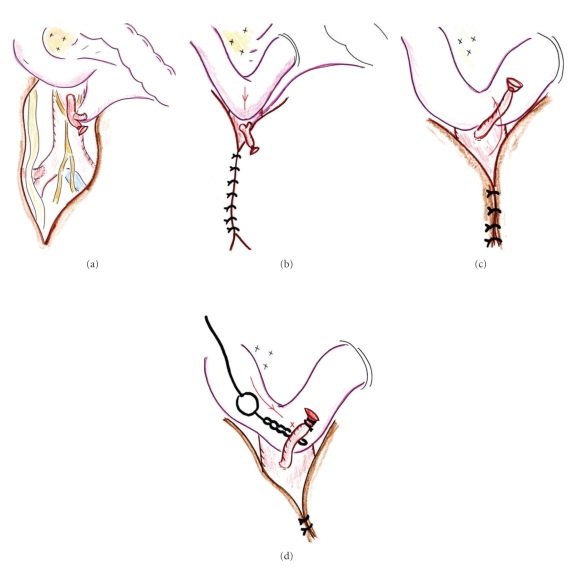
A schematic presentation of the mechanism of bleeding in the present case.

One of the rare points in the present case is the site of bleeding. Delayed postoperative hemorrhage in colorectal surgery has rarely been reported. All reports, including Japanese case reports, have demonstrated that the usual source of hemorrhage is a pelvirectal space associated with pseudoaneurysm of the ramification of the iliac artery and that the mechanism for formation of pseudoaneurysm was violation or exposure of the tunica adventitia of these vessels caused by lymph node dissection or postoperative anastomotic leakage [2–4]. In the present case, the source of hemorrhage was identified at the stump of the SRA. Although postpancreatectomy hemorrhage often occurs via active bleeding from the stump of the GDA, closely associated with anastomotic leakage at the pancreatojejunostomy [5], our search of PUBMED showed no previous reports describing delayed hemorrhage from the stump of the SRA after colorectal surgery. In addition, a characteristic of this uncommon complication is that bleeding from the stump of the SRA was directly linked with the duodenum, leading to intraluminal hemorrhage without expansion into the abdominal cavity. Here we investigate the mechanism of this uncommon complication with a main focus on factors associated with surgical procedures in the present case. First, during lymph node dissection, the IMA was preserved and skeletonized from its base to its tributary (the peripheral side of the root of the LCA). The stump of the SRA was located approximately 4 cm distal to the base of the IMA (Figure 5(a)). Second, through the excision of the mesocolon, the peritoneum was resected from the infraduodenal portion to the bifurcation of the iliac artery, exposing the anterior aspect of the aorta, resulting in thebroad range of peritoneal defect. The peritoneal defect was repaired with an interrupted suture using 3-0 absorbable sutures. Because the transverse defect was quite broad, the suture line of peritoneal reconstruction yielded robust tension and contraction of the adjacent organs. Accordingly, the third portion of the duodenum was displaced downward (Figure 5(b)). Third, during the operation, an end colostomy was constructed with the sigmoid colon. During the maneuver of the left colon, the splenic flexure was not mobilized. Therefore, elevation of the end colostomy toward the abdominal wall yielded spasiticity of the mesenteric root of the left colon, including the stump of the SRA, leading to rotation of the stump of the SRA toward the cephalad portion very close to the anterior aspect of the duodenum (Figure 5(c)). Fourth, unfortunately, the patient developed palalytic ileus at the 3rd postoperative day. The flexion of the duodenum made it difficult to place the decompression tube distal to the ligament of Treitz. We infer that a strong force to the third portion of the duodenum during the insertion of the decompression tube violated the duodenal wall, and the stump of the SRA (Figure 5(d)).

In the present case, the patient developed delayed postoperative hemorrhage 4 days after placement of the decompression tube for postoperative ileus. Although we could not identify the formation of pseudoaneurysm on the emergency CT or angiography, it is assumed that exposure of the tunica adventitia of the IMA and contact between the decompression tube and the stump of the SRA caused a pseudoaneurysm of the stump.

## 3. Conclusion

To avoid this severe complication, we should improve surgical procedures including the reduction of the clearance of peritoneum, expansion of mobilization of the left colon to the splenic flexure, leading to withdrawal of over spasticity in the stump of the SRA, the mesenteric root, and the duodenum.
